# Probucol protects against brain damage caused by intra-neural pyroptosis in rats with vascular dementia through inhibition of the Syk/Ros pathway

**DOI:** 10.18632/aging.205593

**Published:** 2024-02-23

**Authors:** Jingyi Zhu, Jing Du, Wenhui Kou, Chenling Liu, Jianchun Fan, Ziyan Zhu, Lexiu Deng, Lingling Guan, Yuandi Wang, Aimei Yu

**Affiliations:** 1 Graduate School, Hebei North University, Zhangjiakou, Hebei, China; 2 Department of Neurology, The First Affiliated Hospital of Hebei North University, Zhangjiakou, Hebei, China

**Keywords:** vascular dementia, pyroptosis, probucol, spleen tyrosine kinase, oxidative stress

## Abstract

Background: Neuronal injury in chronic cerebral hypoperfusion (CCH) is the main pathogenic factor of vascular dementia (VD). Clinically, there isn’t a drug specifically for VD; instead, the majority of medications used to treat Alzheimer’s disease (AD) are also used to treat VD. Based on the proven anti-inflammatory and antioxidant effects of Probucol, we hypothesized that it may have therapeutic effects on VD, but more research is required to determine its exact mechanism of action.

Methods: *In vivo* experiment: We used SD rats and most commonly used bilateral carotid artery occlusion (2-VO) in VD for modeling. After successful modeling, SD rats were given Probucol 3.5 mg/kg/day for 8 weeks to evaluate the therapeutic effect. *In vitro* experiment: BV-2 microglia of rats were cultured and divided into Control group and Probucol group. Each group was treated with hypoxia-hypoglycemia, hypoxia-hypoglycemia hydrogen peroxide and hypoxia-hypoglycemia hydrogen peroxide Syk inhibitor respectively.

Results: The results of immunofluorescence and Western blot showed that Probucol could significantly improve the cognitive impairment induced by CCH, and the neuronal damage was also attenuated. On the one hand, the underlying mechanism of Probucol was to reduce oxidative stress and cell apoptosis of hippocampal neurons by inhibiting the expression of phosphorylated spleen tyrosine kinase (P-Syk); On the other hand, it exerted a protective effect by reducing NLRP3-dependent cell pyroptosis and inhibiting neuroinflammation induced by microglia activation.

Conclusion: Probucol could reduce oxidative stress and cell apoptosis by inhibiting the Syk/ROS signaling pathway, thereby improving CCH-induced cognitive impairment *in vitro* and *in vivo*.

## INTRODUCTION

Vascular dementia (VD) is a clinical syndrome characterized by cognitive impairments and memory loss mainly caused by vascular risk factors. It has become the second most common form of dementia worldwide, bringing a heavy burden on both the public and healthcare [1]. Chronic cerebral hypoperfusion (CCH) is a vascular factor-induced long-term state of ischemia and hypoxia. Research has suggested that CCH is the primary driver of VD. Primarily, a reduction in the cerebral blood flow (CBF) after CCH leads to the insufficient supply of oxygen and glucose, oxidative stress and neuroinflammation [2, 3], and the activation of apoptosis and pyroptosis pathways [4, 5], thereby accelerating the progression of VD.

Oxidative stress is recognized as a vital risk factor for the occurrence and development of VD. It can cause an imbalance between the ratio of active antioxidant substances and reactive oxygen species (ROS), which can damage neurons and glial cells. The brain has several pathways that produce ROS, with nicotinamide adenine dinucleotide phosphate (NADPH) oxidase playing a significant role. Inhibition of NADPH oxidase has been found to improve the cognitive impairments of CCH rats [6]. Additionally, ROS can activate the apoptosis mechanism to further damage cells [7, 8]. Studies [9] have also shown that pyroptosis, a form of pro-inflammatory programmed cell death, is involved in the pathological process of vascular cognitive impairments. The NOD-like receptor thermal protein domain associated protein 3 (NLRP3) inflammasome is a multiprotein complex composed of NLRP3, apoptosis-associated speck-like protein containing a CARD (ASC), and pro-cysteinyl aspartate specific proteinase-1 (pro-Caspase-1) [10]. By activating caspase-1, the NLRP3 inflammasome mediates pyrolysis, triggering the innate immune response. Microglial cells, the only innate immune cells in the central nervous system, are the earliest responders to brain injury. While transient neuroinflammation is beneficial for microglial cells to maintain brain homeostasis [11], long-term inflammatory stimulation can result in the overactivation of microglial cells, exacerbating neuronal death [12]. This may be due to the inflammasome being the main responder to microglial cell-mediated neuroinflammation. Therefore, targeting pyroptosis is a potential strategy for preventing VD.

The mechanisms of NLRP3 inflammatory vesicle activation include K^+^ efflux, histone B release and ROS production [13]. While the molecular mechanisms of NLRP3 inflammatory vesicle activation are still being determined, recent studies suggest that the upstream spleen tyrosine kinase (Syk) is a key mediator of the process. Syk can participate in signal transduction in various cell types and is a critical mediator of immune receptor signaling in inflammatory cells in the body [14]. Studies [15] have demonstrated that Dectin-1 activation can contribute to Syk-induced ROS triggering the assembly of NLRP3 inflammatory vesicles. However, the interaction between Syk and NLRP3 in CCH-induced neuroinflammation remains to be elucidated.

Probucol has anti-inflammatory, antioxidant and hypolipidaemic effects and is most commonly used clinically for the reduction of cholesterol and atherosclerosis [16]. Some studies have shown that probucol also has some neuroprotective effects, ameliorating neuronal damage due to cerebral ischaemia by inhibiting lipopolysaccharide-induced microglial activation [17], and reducing streptozotocin-induced cognitive impairment and biochemical changes in the hippocampus [18].

This study was designed to investigate the possible mechanism of probucol in improving cognitive impairments in the VD rat model, which is expected to provide some evidence for probucol as a potential treatment for VD.

## MATERIALS AND METHODS

### Animals and grouping

52 SPF-grade male Sprague-Dawley (SD) rats (SPF (Beijing) Biotechnology Co., Ltd., China), weighing 200–220 g, were housed in a clean animal room with a temperature of (24 ± 2°C), a relative humidity of 40-60%, and a 12:12-h light/dark cycle. The design and implementation of the animal experiments were approved by the Animal Ethics Committee of Hebei North University. The ethics number is HBNU20221207223043.

### CCH rat model and experimental design

Bilateral common carotid artery occlusion is the major cause of CCH, so the Surgical Method 1 described in previous studies was adopted here. To establish the model, the rats were first injected with 1% pentobarbital sodium into the abdomen for general anesthesia. An abdominal midline incision was made in the neck to expose the bilateral common carotid arteries. The right common carotid artery was ligated, at 30 min after which the left common carotid artery was ligated. Then the rat wound was sutured to complete the model establishment. The control group was rats without surgery. In sham group, the bilateral common carotid arteries were not ligated, and the rest of the procedures were the same as above. The rats were randomly divided into control group (*n* = 12), sham group (*n* = 12), CCH group (*n* = 12), and probucol (3.5 mg/kg/day) [19] group (*n* = 12). The rats in probucol group received intragastrical administration of probucol (Sigma, St. Louis, MO, USA) at 3.5 mg/kg/day, and those in sham and CCH groups received the same volume of distilled water as the rats in probucol group for eight consecutive weeks.

### Morris water maze test

After modeling, the Morris water maze test was adopted to assess and analyze cognitive function. The test consisted of two parts: (1) Orientation and navigation test: The three groups of rats were placed into the maze for 2 min to familiarize themselves with the environment. They were then placed in the pool from each of the four quadrants with their heads facing the wall, and the time it took for the rats to climb onto the platform within 60 s was recorded as the escape latency. If the rats failed to find the platform within 60 s, the escape latency time would be recorded as 60 s. The rats were trained for five consecutive days. (2) Space exploration test: On the 6th day, the platform was removed, and the rats were placed on the opposite side of the quadrant where the original platform quadrant was located. The frequency of crossing the target quadrant platform, the percentage of residence time in the quadrant, and the average swimming speed were recorded and analyzed based on their 60-s trajectories.

### Open field experiments

Rats were placed in observation boxes (80 cm × 80 cm × 40 cm). The walls and floor are black. The floor is divided into 9 rectangles, with the middle rectangle defined as the center. It is carried out in a well-lit and well-temperature room (constant temperature, 22 ± 2°C). Animal behavior tracking systems were used (EthoVision 3.0; Noldus Information Technology, Wageningen, The Netherlands). Adaptive activities were performed for 2 min, and then the number of trajectories running through the center, the time in the center, the rat travel distance, and the number of hind leg stands were recorded.

### TUNEL

Assess apoptosis using the TUNEL kit according to the manufacturer's instructions. Briefly, TUNEL-positive cells are observed under a microscope. Apoptotic cells were counted using ImageJ software, and 5 fields of view were randomly selected and observed under a fluorescence microscope. The apoptosis index (AI) is determined by the following formula: AI = number of positive cells in each field/total number of cells in each field × 100%.

### Hematoxylin and eosin (HE) staining

After deep anesthesia with an intraperitoneal injection of 1% pentobarbital sodium (45 mg/kg. ip), the heart harvested was perfused with 0.9% NaCl, fixed with 4% paraformaldehyde overnight, embedded in paraffin, and sectioned (4 μm). Following deparaffinization and dehydration, the sections were stained with hematoxylin and eosin for 4 min and 30 s, respectively. The morphological changes of hippocampal neurons were observed under an optical microscope (Lecia DM750, Japan) at the magnifications of 100× and 400×, respectively.

### Immunofluorescence

According to standard histological procedures, the sections were pre-incubated with 5% goat serum for 30 min at room temperature. The sections were then incubated with rabbit polyclonal anti-Iba1 antibody (1:100, ab178847, ab195032). Later, the sections were incubated with Alexa Flour^®^ 488 (ab150077, Abcam, UK) and Alexa Flour^®^ 647 (ab150075, Abcam) secondary antibodies. After the sections were washed with phosphate-buffered saline (PBS) three times, 4′,6-diamidino-2-phenylindole (DAPI, D9542, Sigma-Aldrich) was added for staining. Subsequently, immunofluorescence data were used for quantitative analysis using an Olympus microscope to detect the activation of hippocampal microglial cells.

### Cell culture and grouping

The murine microglial cell line BV2 was bought from Procell Life Science and Technology Co., Ltd., Wuhan, China. BV2 cells were inoculated in the Minimum Essential Medium (MEM) containing 10% fetal bovine serum (Sigma-Aldrich) and 1% penicillin/streptomycin (Beyotime Institute of Biotechnology, Shanghai, China) and cultured in an incubator at 37°C and 5% CO_2_. The oxygen-glucose deprivation model was established by culturing BV2 cells in the low-glucose MEM (41090036, Thermo Fisher Scientific, USA) in a tri-gas incubator (Thermo Forma 3111) at 37°C, 1% O_2_, 5% CO_2_, and 94% N_2_. The cells were divided into Control group and probucol (10μM) group and then treated with low oxygen + low glucose (LO/LG), low oxygen + low glucose + hydrogen peroxide (30 μM) (LO/LG/H_2_O_2_), and low oxygen + low glucose + hydrogen peroxide + SYK inhibitor (BAY61-3606, Selleck, USA) (LO/LG/H_2_O_2_/BAY), respectively.

### Western blotting (WB) assay

The total protein was extracted from brain tissues and BV2 microglial cells using RIPA lysis buffer (Beyotime Institute of Biotechnology), and its concentration was determined using a BCA protein assay kit (Beyotime Institute of Biotechnology). Then the proteins were separated on an SDS-PAGE gel (8–12%) and transferred onto a PVDF membrane. The membrane was incubated in 5% skimmed milk in TBST for 1 h at room temperature, followed by incubation with the corresponding primary antibodies at 4°C overnight. The primary antibodies used were as follows: SYK (1:1000, ab40781), p-Syk (1:500, SAB4300282-100UG), gp91phox (1:5000, ab129068), p47phox (1:500, SAB4504721-100UG), p22phox (1:500, SAB4504721-100UG), p22phox (1:500, SAB4504721-100UG), 1000, 37570), NLRP3 (1:500, ab263899), ASC (1:1000, ab307560), Caspase-1 (1:1000, ab179515), B-cell lymphoma 2 (Bcl-2, 1:500, ab196495), Bcl-2-assocaited X protein (Bax, 1:1000, ab32503), Caspase-3 (1:2000, ab184787). The membrane was then incubated with anti-rabbit secondary antibodies for 2 h at room temperature, and the protein bands were visualized using enhanced chemiluminescence. Finally, the gray values of bands were analyzed using ImageJ software (Millipore, Burlington, MA, USA), and β-actin was used as an internal reference for comparative analysis.

### Flow cytometry

Cells were cultured in six-well plates and exposed to different conditions in different groups. After exposure, the cells were trypsinized, centrifuged, and washed three times with cold PBS. Next, the cells were resuspended in 100 μL of binding buffer, followed by a reaction with 5 μL of Annexin V-fluorescein isothiocyanate (FITC) and 5 μL of propidium iodide (PI) at room temperature for 15 min in the dark. Finally, the cells were mixed gently with 400 μL of binding buffer, and subjected to flow cytometry (CytoFLEX LX, Indianapolis, IN, USA).

### Statistical methods

The normality and homogeneity of variance of the data were tested using GraphPad Prism 9.0 software. Measurement data that were normally distributed were expressed as mean ± standard deviation. One-way analysis of variance was used for comparison among groups, and the *t*-test was adopted for comparison between two groups. *P* < 0.05 indicated that the difference was statistically significant.

## RESULTS

### Probucol improved the learning and memory ability of rats with VD

**Figure 1:**
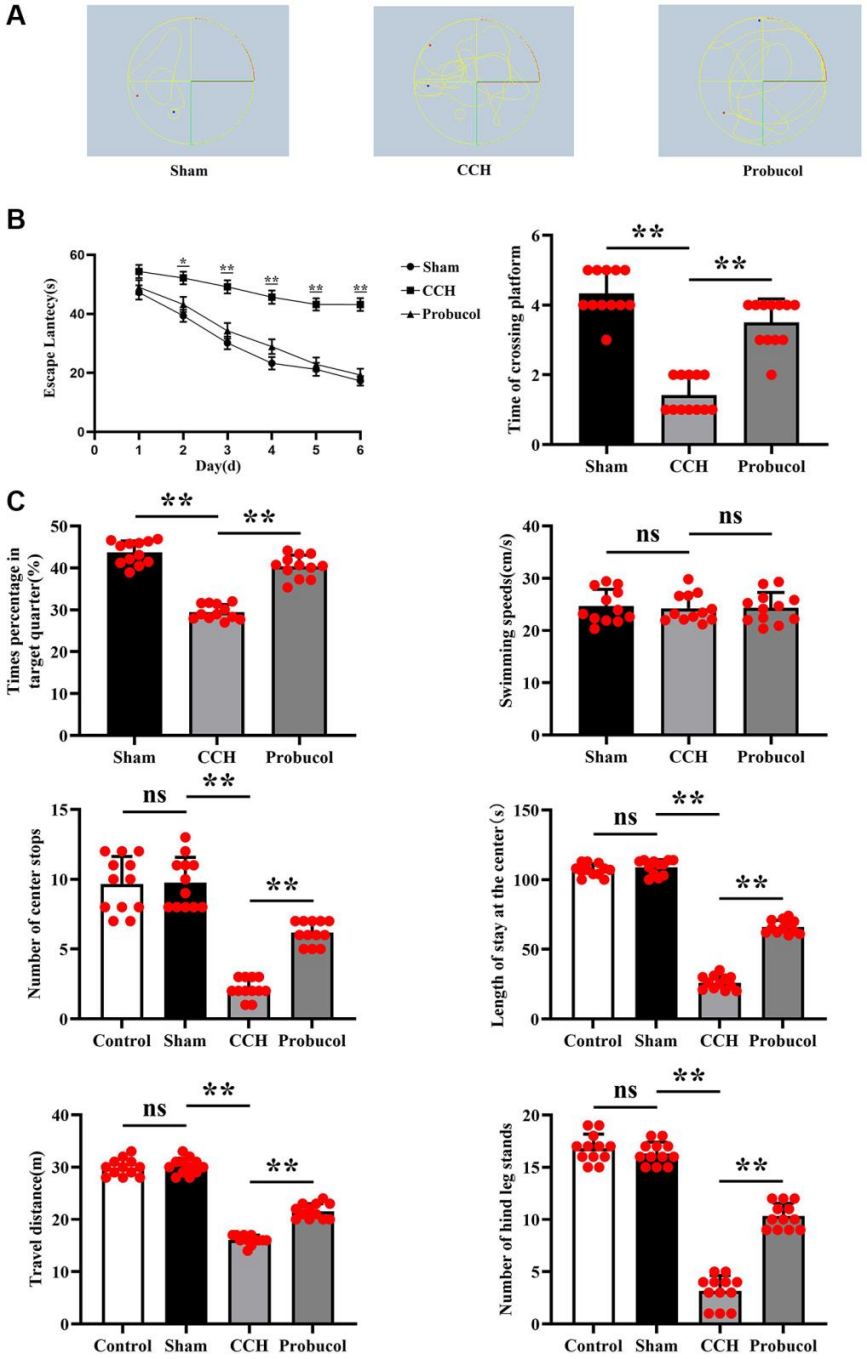
**Probucol improves CCH-induced spatial learning and memory.** (**A**) Path map of the Morris water maze in sham group, CCH group, and probucol group. (**B**) Escape latencies of rats in the hidden platform task; Frequency of crossing the platform in the space exploration test; Percentage of residence time in the target quadrant during the space exploration test; Average swimming speed of rats in the visible platform test. (**C**) Statistics on the number of center crossings in the control group, sham operation group, CCH group and probucol group; Statistics of time in the center of the control group, sham group, CCH group and probucol group; Travel distance statistics of control group, sham operation group, CCH group and probucol group; Standing time statistics of rats in the control group, sham operation group, CCH group and probucol group. Data are expressed as mean ± standard error of mean (SEM). *n* = 12/group. ^**^P < 0.01: CCH group vs. Probucol group.

In the Morris water maze experiment, 48 male SD rats were included. Two of the rats died after modelings. The remaining 46 rats all completed this experiment. After our calculations, the success rate of rat modeling was 91.6%, and the remaining two rats were excluded from this experiment. The results in the hidden platform test show (*P* < 0.05), The CCH group exhibited significantly prolonged avoidance latencies compared to the Sham group, and administration of Probucol treatment was found to significantly improve spatial learning memory due to CCH (29.78 ± 11.57; 47.97 ± 4.71; 32.94 ± 11.56); (Sham VS CCH, *P* < 0.01); (CCH VS Probucol, *P* < 0.01). In the space exploration test, the rats in CCH group exhibited fewer times of crossings the original platform (4.33 ± 0.65; 1.42 ± 0.51; 3.50 ± 0.67); (Sham VS CCH, *P* < 0.01); (CCH VS Probucol, *P* < 0.01), decreased residence time in the target quadrant (43.68 ± 2.77; 29.48 ± 1.80; 40.33 ± 2.72); (Sham VS CCH, *P* < 0.01); (CCH VS Probucol, *P* < 0.01), and a chaotic trajectory in contrast to sham group. Nevertheless, these spatial memory impairments were notably alleviated following probucol treatment (Figure 1, *P* < 0.01). The average swimming speed showed no statistically significant difference (24.69 ± 3.20; 24.25 ± 2.69; 24.37 ± 2.92); (Sham VS CCH, *P* = 0.7214); (CCH VS Probucol, *P* = 0.9223). We then tested the cognitive conditioning in rats by an open field experiment. And we added rats before the surgical intervention. The results showed that there were no significant differences in the number of times the rats crossed the center (9.67 ± 1.97; 9.75 ± 1.82; 2.17 ± 0.72; 6.17 ± 0.83); (Control VS Sham, *P* = 0.9152); (Sham VS CCH, *P* < 0.01); (CCH VS Probucol, *P* < 0.01), the time they stood in the center (106.8 ± 4.43; 108.9 ± 5.40; 26.08 ± 4.91; 66.08 ± 4.76); (Control VS Sham, *P* = 0.3127); (Sham VS CCH, *P* < 0.01); (CCH VS Probucol, *P* < 0.01), the travel distance (29.92 ± 1.62; 30.17 ± 1.53; 16.08 ± 0.90; 21.58 ± 1.44); (Control VS Sham, *P* = 0.7012); (Sham VS CCH, *P* < 0.01); (CCH VS Probuco, *P* < 0.01) and the number of times they stood on the hind legs (16.83 ± 1.34; 16.33 ± 1.07; 3.17 ± 1.47; 10.33 ± 1.23); (Control VS Sham, *P* = 0.3234); (Sham VS CCH, *P* < 0.01); (CCH VS Probucol, *P* < 0.01) between the control group and the Sham group. Compared with the control group and the sham group, the number of times the rats crossed the center, the time they stood in the center, the travel distance and the number of hind legs standing in the CCH group and the Probucol group were significantly reduced. Compared with the CCH group, the number of times the rats crossed the center, the time they stood in the center, the travel distance and the number of hind legs standing in the Probucol group were significantly increased (Figure 1). To sum up, the results indicated that probucol significantly mitigates spatial learning and memory impairments caused by CCH.

### Probucol attenuated CCH-induced neuronal damage

**Figure 2:**
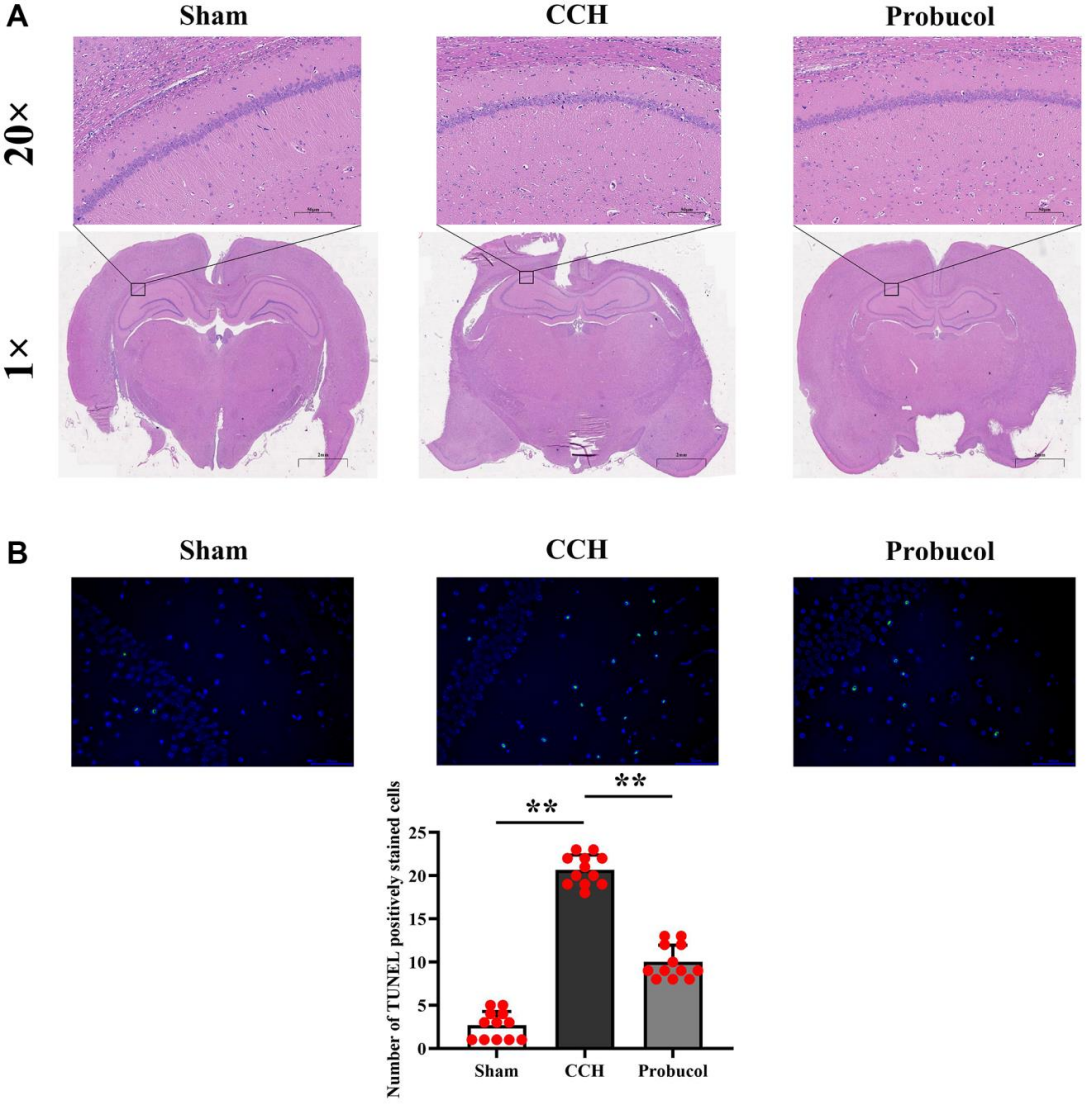
**Probucol attenuates CCH-induced neuronal death in the hippocampus.** (**A**) Sham, CCH, Probucol group respectively Hippocampal slices are stained with HE (scale bar = 50 μm or 2 mm). (**B**) TUNEL staining results and the number of apoptotic cells.

Figure 2 shows the representative photomicrographs of neurons in the hippocampal CA1 region. In sham group, neurons in the hippocampal CA1 region were regularly and compactly arranged with well-distributed cytoplasmic staining. In contrast, CCH group showed neuronal loss, shrinkage, and loose arrangement in these regions. However, compared with the CCH group, neuronal damage in the CA1 region of the hippocampus was partially reduced in the Probucol group. We used the TUNEL staining experiment and found that the number of apoptotic cells in the CCH and probucol groups was significantly increased compared to sham groups, while the number of apoptotic cells in the probucol group was significantly reduced compared to the CCH group. The number of active neurons in the hippocampus CA1 region in CCH group was remarkably smaller than that in probucol group (2.67 ± 1.61; 20.67 ± 1.72; 10.00 ± 1.95, *P* < 0.01).

### Probucol suppressed CCH-induced oxidative stress in the hippocampus

**Figure 3:**
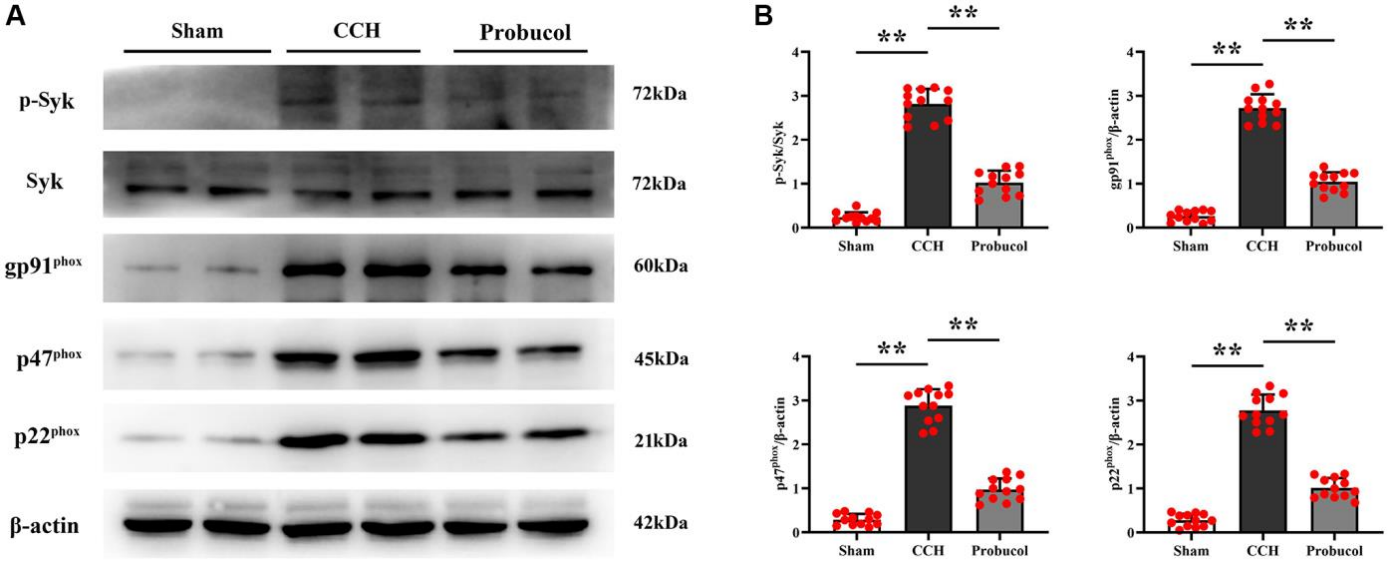
**Probucol reduces CCH-induced oxidative stress.** (**A**) WB assay of p-Syk, Syk, and oxidative stress markers (gp91phox, p47phox, and p22phox). (**B**) Statistical data of WB assay results. Data are expressed as mean ± SEM. *n* = 12/group. ^**^P < 0.01: CCH group vs. Probucol group.

The role of oxidative stress in the progression of cognitive impairments in VD has been well recognized. To investigate the protective mechanism of probucol against cognitive impairments in CCH rats and to further explore the effect of probucol on hippocampal oxidative damage in CCH rats, we analyzed by western blot in the rat hippocampus of Syk, p-Syk, gp91^phox^, p47^phox^, and p22^phox^ (Figure 3). The results showed that the protein expression levels of p-Syk (0.24 ± 0.11; 2.82 ± 0.34; 1.03 ± 0.27), gp91^phox^ (0.26 ± 0.12; 2.72 ± 0.31; 1.05 ± 0.22), p47^phox^ (0.29 ± 0.13; 2.88 ± 0.37; 0.97 ± 0.25) and p22^phox^ (0.28 ± 0.14; 2.78 ± 0.35; 1.01 ± 0.23) in the CCH group and the probucol group were significantly increased compared with the sham group. Compared with the CCH group, the protein expression levels of p-Syk (Sham VS CCH, *P* < 0.01); (CCH VS Probucol, *P* < 0.01), gp91^phox^ (Sham VS CCH, *P* < 0.01); (CCH VS Probucol, *P* < 0.01), p47^phox^ (Sham VS CCH, *P* < 0.01); (CCH VS Probucol, *P* < 0.01) and p22^phox^ (Sham VS CCH, *P* < 0.01); (CCH VS Probucol, *P* < 0.01) in the probucol group were significantly reduced. These findings suggest that probucol may alleviate hippocampal oxidative stress in CCH rats by reducing p-Syk.

### Probucol inhibited CCH-induced hippocampal neuroinflammation in rats

**Figure 4:**
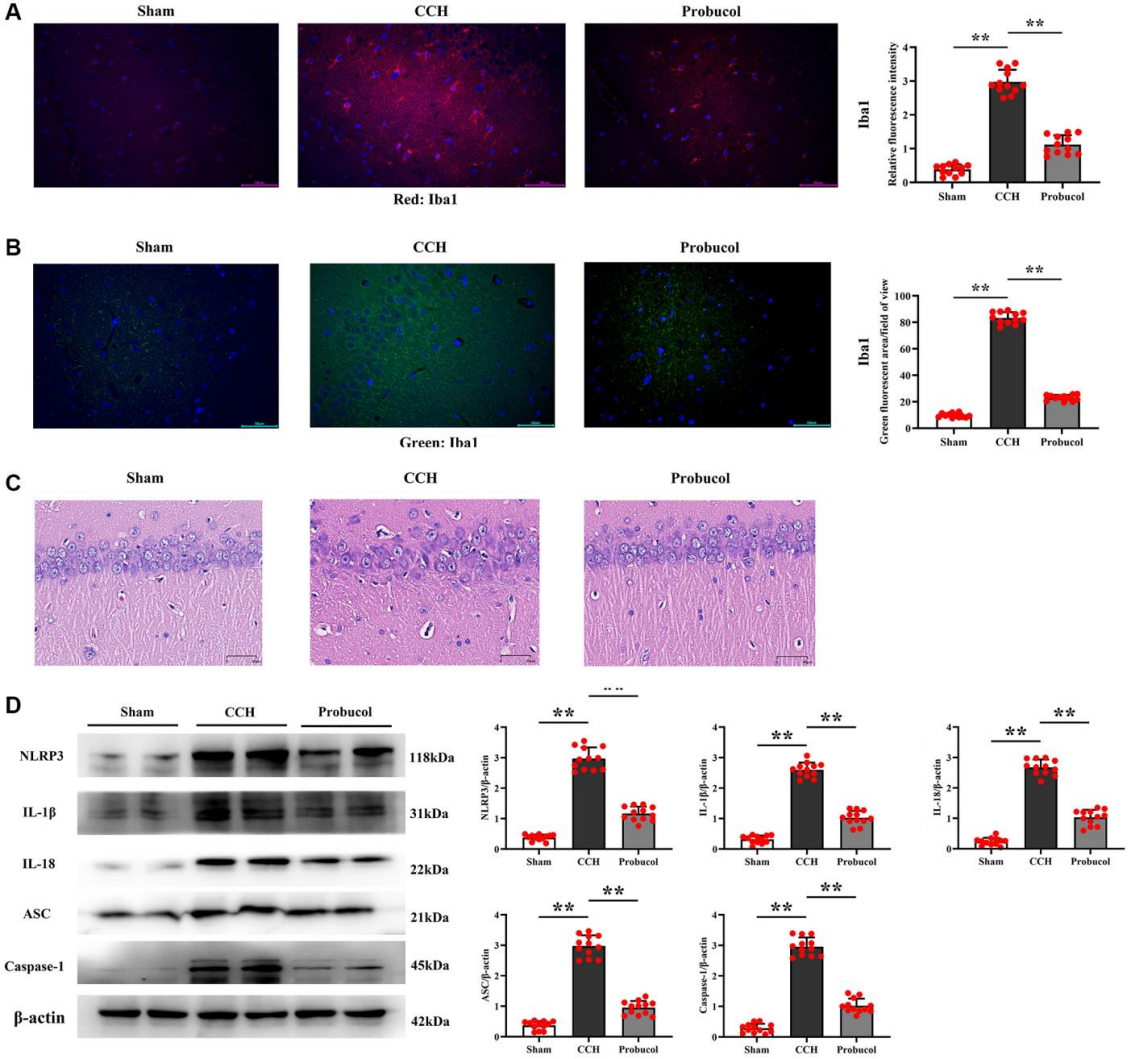
**Probucol reduces CCH-induced microglial activation and pyroptosis.** (**A**, **B**) Immunofluorescence of Iba1/DAPI. Representative photomicrographs of microglial activation in the Hippocampal region (scale bar = 50 μm). (**C**) Pyramidal cell layer HE staining results (scale bar = 40 μm). (**D**) WB assay of pyroptotic markers (NLRP3, ASC, and Caspase-1) and inflammatory cytokines (IL-1β and IL-18). Data are expressed as mean ± SEM. *n* = 12/group. ^**^P < 0.01: CCH group vs. Probucol group.

Neuroinflammation is known to play a crucial role in the occurrence and development of VD. Microglial cells, the brain's resident immune cells, are critical in initiating and transmitting inflammatory responses, with inflammasomes being the primary responders to microglial cell-mediated neuroinflammation. Therefore, to examine the effect of probucol on NLRP3 inflammasome in microglial cells, hippocampal slices were subjected to immunofluorescent staining with Iba1 as a specific marker for microglial cells. As shown in Figure 4A–4C, the relative fluorescence intensity of Iba1 in the hippocampus of the CCH group was significantly higher than that of the sham group (0.38 ± 0.15; 2.98 ± 0.36; 1.12 ± 0.28); (Sham VS CCH, *P* < 0.01); (CCH VS Probucol, *P* < 0.01). Compared with the CCH group, the relative fluorescence intensity of Iba1 in the probucol group was significantly reduced (9.66 ± 1.54; 83.39 ± 4.33; 22.99 ± 2.16); (Sham VS CCH, *P* < 0.01); (CCH VS Probucol, *P* < 0.01). There were fewer activated microglia in the hippocampus of the sham group and more Iba1+ cells in the hippocampus in the CCH group. The results of HE staining showed that probucol could alleviate the neuronal damage caused by CCH. In addition, in the probucol group, hyperactivation of microglia was inhibited.

Furthermore, a WB assay was performed, as illustrated in Figure 4D. The results revealed that compared with sham group, CCH group and probucol group exhibited increased levels of IL-1β (0.32 ± 0.13; 2.60 ± 0.24; 1.03 ± 0.23), IL-18 (0.24 ± 0.12; 2.68 ± 0.25; 1.03 ± 0.25), and pyroptosis-associated inflammasome proteins (NLRP3 (0.38 ± 0.10; 2.97 ± 0.37; 1.12 ± 0.23), ASC (0.37 ± 0.14; 2.98 ± 0.35; 0.95 ± 0.22), and Caspase-1 (0.29 ± 0.15; 2.96 ± 0.30; 1.02 ± 0.23)). Compared with the CCH group, the protein expression levels of IL-1β (Sham VS CCH, *P* < 0.01); (CCH VS Probucol, *P* < 0.01), IL-18 (Sham VS CCH, *P* < 0.01); (CCH VS Probucol, *P* < 0.01) and pyroptosis-associated inflammasome proteins NLRP3 (Sham VS CCH, *P* < 0.01); (CCH VS Probucol, *P* < 0.01), ASC (Sham VS CCH, *P* < 0.01); (CCH VS Probucol, *P* < 0.01) and Caspase-1 (Sham VS CCH, *P* < 0.01); (CCH VS Probucol, *P* < 0.01) were significantly reduced in the probucol group. However, probucol treatment evidently reduced the CCH-induced inflammatory response. As a result, the levels of pro-inflammatory cytokines and pyroptosis-associated inflammasome proteins were markedly reduced in probucol group compared with those in CCH group.

### Probucol inhibited CCH-induced apoptosis in rats and BV2 cells treated with low oxygen and low glucose

**Figure 5:**
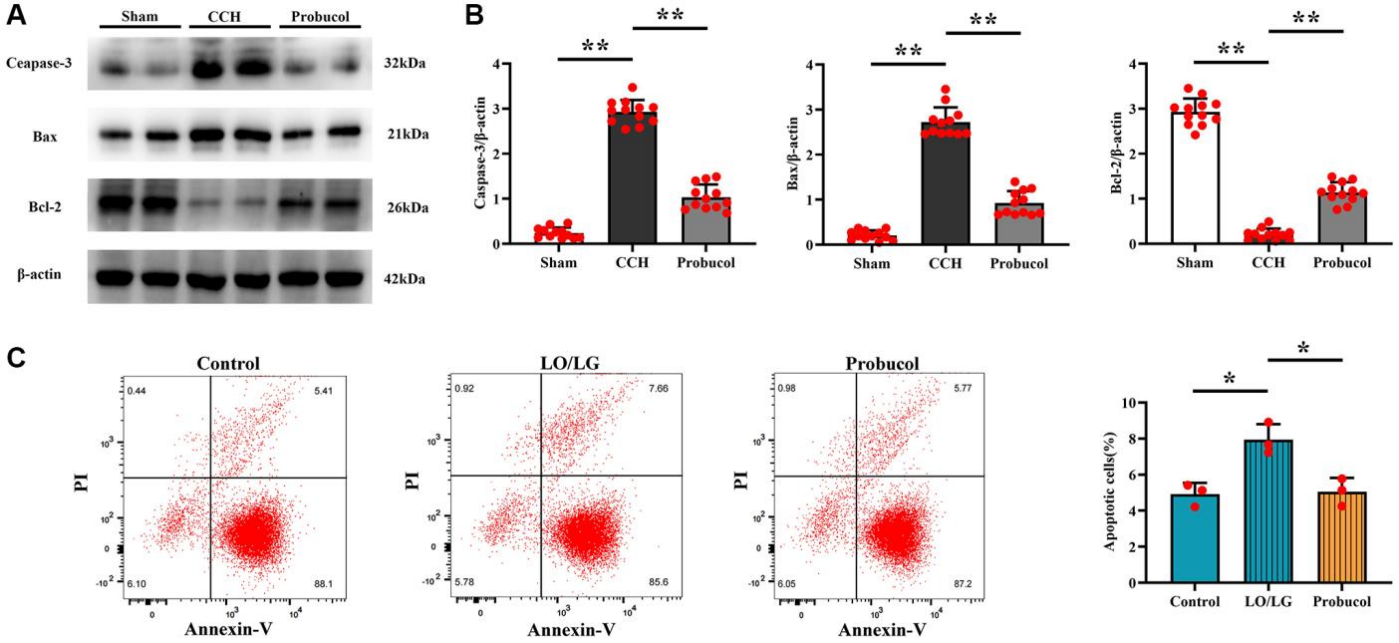
**Probucol attenuates CCH-induced microglial apoptosis in rats and BV2 cells treated with LO/LG.** (**A**) WB assay of apoptosis markers (Caspase-3, Bax, and Bcl-2). (**B**) Statistical data of WB assay results. *n* = 12/group. (**C**) Number of apoptotic cells measured by flow cytometry. *n* = 3/group. Data are expressed as mean ± SEM. *n* = 12/group. ^*^P < 0.05 and ^**^P < 0.01: CCH group vs. Probucol group.

Apoptosis plays a role in the development of VD. In this study, therefore, western blot was employed to assess neuronal apoptosis *in vivo*, and Annexin V-FITC/PI double staining was performed for the detection of microglial cell death *in vitro*. As shown in Figure 5A, 5B, compared with the sham group, the expression levels of Bax (0.21 ± 0.11; 2.72 ± 0.33; 0.92 ± 0.27); (Sham VS CCH, *P* < 0.01); (CCH VS Probucol, *P* < 0.01) and Caspase-3 (0.24 ± 0.12; 2.93 ± 0.26; 1.03 ± 0.28) (Sham VS CCH, *P* < 0.01); (CCH VS Probucol, *P* < 0.01) were increased in the CCH group and the probucol group, while the expression level of Bcl-2 (2.93 ± 0.30; 0.21 ± 0.13; 1.14 ± 0.23); (Sham VS CCH, *P* < 0.01); (CCH VS Probucol, *P* < 0.01) was decreased. However, compared with those in CCH group, the expression levels of Bax and Caspase-3 were down-regulated, while the expression level of Bcl-2 was increased in probucol group.

As revealed by Annexin V-FITC/PI double staining, probucol effectively suppressed the apoptosis of microglial cells induced by oxygen-glucose deprivation (Figure 5C). (4.92 ± 0.63; 7.93 ± 0.87; 5.05 ± 0.76) P (Control VS CCH) = 0.008; (LO/LG VS Probucol, *P* < 0.01). Furthermore, the results of *in vitro* experiments were consistent with those of *in vivo* experiments.

### Probucol protected BV2 microglial cells against oxidative stress and pyroptosis by inhibiting p-Syk

**Figure 6:**
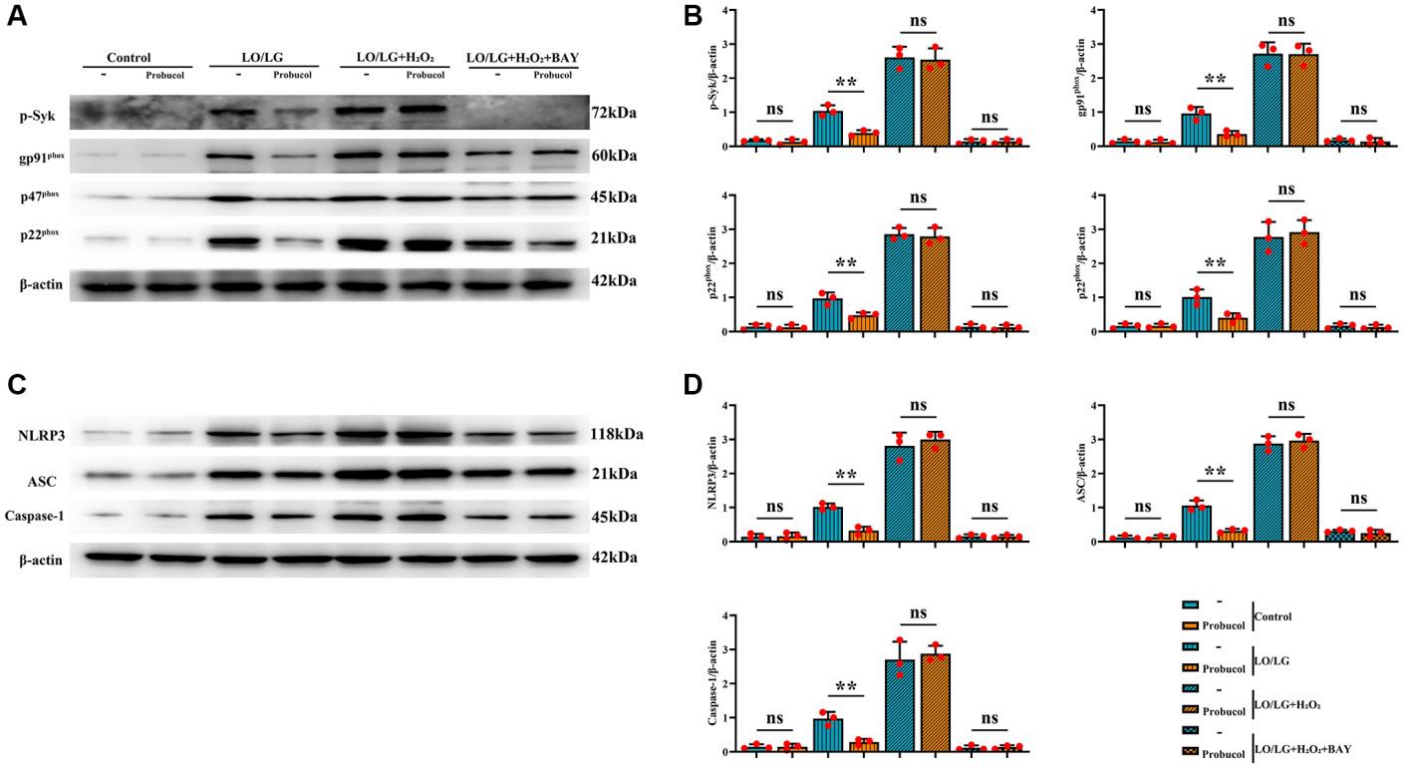
**Probucol reduces oxidative stress and pyroptosis in microglial cells treated with LO/LG.** (**A**) WB assay of p-Syk and oxidative stress markers (gp91^phox^, p47^phox^, and p22^phox)^. (**B**) Statistical data of WB assay results. (**C**) WB assay of pyroptosis markers (NLRP3, ASC, and Caspase-1). (**D**) Statistical data of WB assay results. Data are expressed as mean ± SEM. *n* = 3/group. ^**^P < 0.01: LO/LG group vs. Probucol group.

For the evaluation of the potential role of p-Syk (0.18 ± 0.03; 0.13 ± 0.08; (*P* = 0.3987) 1.04 ± 0.16; 0.39 ± 0.08; (*P* = 0.0036) 2.61 ± 0.31; 2.54 ± 0.33; (*P* = 0.8038) 0.15 ± 0.07; 0.15 ± 0.07 (*P* = 0.9542)) in the protective effect of probucol against oxidative stress and NLRP3 inflammasome activation, the effect of probucol on p-Syk was investigated. First, probucol was demonstrated to exert no effect on p-Syk under normal conditions (Figure 6). However, probucol evidently decreased the expression of p-Syk in LO/LG group. Additionally, the WB assay revealed that the expression levels of gp91^phox^ (0.14 ± 0.06; 0.13 ± 0.06; (*P* = 0.7597) 0.96 ± 0.19; 0.36 ± 0.09; (*P* = 0.0072) 2.72 ± 0.33; 2.70 ± 0.30; (*P* = 0.9615) 0.18 ± 0.04; 0.14 ± 0.10 (*P* = 0.5645)), p47^phox^ (0.14 ± 0.08; 0.13 ± 0.07; (*P* = 0.8057) 0.97 ± 0.18; 0.48 ± 0.09; (*P* = 0.0121) 2.86 ± 0.18; 2.79 ± 0.25 (*P* = 0.7245) 0.14 ± 0.08; 0.12 ± 0.08; (*P* = 0.8077)), p22^phox^ (0.17 ± 0.07; 0.17 ± 0.06; (*P* > 0.05) 1.01 ± 0.06; 0.41 ± 0.13; (*P* = 0.0162) 2.77 ± 0.44; 2.91 ± 0.36; (*P* = 0.6906) 0.17 ± 0.07; 0.14 ± 0.06; (*P* = 0.5823)), NLRP3 (0.15 ± 0.09; 0.16 ± 0.11; (*P* = 0.8430) 1.02 ± 0.10; 0.33 ± 0.11; (*P* = 0.0012) 2.81 ± 0.39; 2.99 ± 0.23; (*P* = 0.5251) 0.16 ± 0.06; 0.15 ± 0.05; (*P* = 0.8798)), ASC (0.13 ± 0.06; 0.13 ± 0.06; (*P* = 0.9473) 1.06 ± 0.06; 0.32 ± 0.06; (*P* = 0.0014) 2.88 ± 0.22; 2.96 ± 0.20; (*P* = 0.6343) 0.31 ± 0.04; 0.25 ± 0.10; (*P* = 0.3672)), and Caspase-1 (0.15 ± 0.07; 0.15 ± 0.09; (*P* = 0.9622) 0.98 ± 0.20; 0.29 ± 0.09; (*P* = 0.0052) 2.70 ± 0.53; 2.87 ± 0.23; (*P* = 0.6360) 0.12 ± 0.07; 0.14 ± 0.05; (*P* = 0.6754)) were significantly reduced in LO/LG group after probucol treatment.

**Figure 7:**
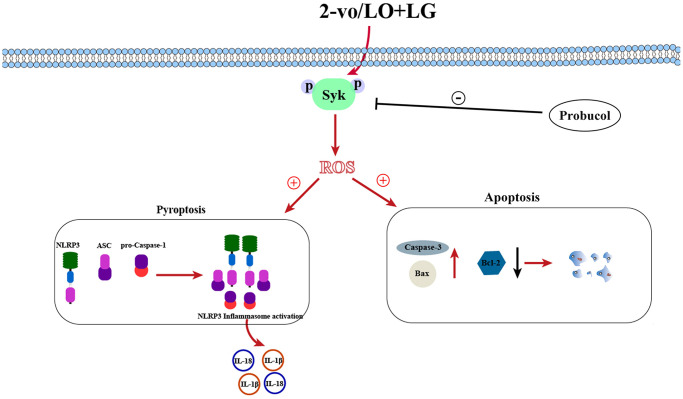
Schematic diagram of probucol improving vascular cognitive impairment.

Afterward, whether the elevation of p-Syk induced by LO/LG/H_2_O_2_ affects the reduction of oxidative stress and NLRP3 inflammasome activation by probucol was investigated. In LO/LG/H_2_O_2_ group, the expression levels of gp91^phox^, p47^phox^, p22^phox^, NLRP3, ASC, and Caspase-1 were significantly elevated, which, however, were not changed by probucol. Furthermore, we found that the addition of Syk inhibitor in LO/LG/H_2_O_2_ group intervened with the addition of Probucol in LO/LG/H_2_O_2_, the protein expressions of p-Syk, gp91^phox^, p47^phox^, p22^phox^, NLRP3, ASC, and Caspase-1 declined, and there were no statistical differences between the two groups. The above results indicated that probucol protects BV2 cells against pyroptosis and oxidative stress by reducing the protein expression of p-Syk. In summary, probucol treatment can inhibit the expression of p-Syk, thereby reducing microglial activation, pyroptosis, and ROS production in the hippocampus of VD mice (Figure 7). The raw data of this study are provided as Supplementary Table 1.

## DISCUSSION

Probucol contains two easily oxidizable phenolic hydroxyl groups that can form stable phenolic oxygen groups by binding to oxygen free radicals, exhibiting potent effects such as lipid-lowering, anti-inflammatory, and anti-oxidative properties. There is increasing evidence that probucol has therapeutic potential for neurodegenerative diseases [20, 21]. However, it is not clear whether probucol exerts a protective effect against VD by affecting Syk. Therefore, to investigate whether Probucol plays a pathological protective role against VD by affecting Syk. Stable models were established *in vivo* and *in vitro*, We used the most commonly used bilateral carotid occlusion method (2-VO) for CCH and VD to establish an *in vivo* animal model. *In vitro*, a corresponding model was established by exposing BV2 microglial cells to a LO/LG environment.

2-VO-induced CCH can cause neuronal damage through oxidative stress and neuroinflammation, exacerbating learning and memory impairment in rats [2, 3]. Therefore, to evaluate the effect of Probucol on the learning and memory functions of CCH rats, we used the Morris water maze test and the open field test. In the Morris water maze test, the escape latency of rats in the CCH group was prolonged, and they could cross the original platform. The number of repetitions and the time spent in the target quadrant were reduced, which is consistent with previous research [22]. However, long-term Probucol treatment can significantly improve spatial learning and memory impairment in CCH rats. In the Open field experiment, CCH group rats showed less anxiety and better exploration ability after Probucol treatment. In addition, we also found that Probucol alleviated CCH-induced neuronal damage, HE staining showed a decrease in withered neurons, and TUNEL staining showed a decrease in apoptotic cells. These results are consistent with our behavioral consequences in the Morris water maze and Open field experiments. The results are consistent. These findings suggest that Probucol has a protective effect against cognitive impairment in CCH rats.

The brain contains a high concentration of polyunsaturated fatty acids, which are susceptible to oxidation by ROS, and also has a high concentration of iron which acts as an antioxidant [23]. In CCH-induced VD, ROS is primarily produced by NADPH oxidase, which is a multi-subunit complex composed of cytoplasmic subunits (p47^phox^, p67^phox^, and p40^phox^) and membrane subunits (gp91^phox^, p22^phox^, and Rac) [24]. During CCH, the expression of NADPH oxidase subunits is elevated, which is associated with cognitive impairments [24]. A study has shown that the expression levels of gp91^phox^ and p47^phox^ were remarkably raised in the microglial cells and neurons of patients with mild cognitive impairments [25]. p47^phox^ and p22^phox^ can affect the activity of NADPH oxidase. However, few reports have examined the changes in NADPH subunit expression after CCH. In this study, significant increases in the expression levels of gp91^phox^, p47^phox^, and p22^phox^ were observed in CCH group, and significant decreases were observed in probucol group.

ROS is crucial for apoptosis, and apoptosis plays a critical role in VD. ROS can activate DNA repair enzymes and generate poly (ADP-ribose), which is recognized by neurons as an apoptotic signal. Poly (ADP-ribose) binding to apoptosis-inducing factor can activate Caspase-3, leading to apoptosis [8]. The Bax/Bcl-2/Caspase-3 signaling pathway, which includes Bax as a pro-apoptotic member of the Bcl-2 protein family and Bcl-2 as an anti-apoptotic protein, has been extensively studied. ROS can cause the dysregulation of apoptotic pathways, resulting in cell death and promoting disease progression [26]. This study uncovered that significant neuronal damage was caused in the hippocampus of rats in CCH group, and probucol could significantly improve neuronal damage in the hippocampus. Additionally, the expression levels of Caspase-3 and Bax were significantly elevated, but the expression level of Bcl-2 obviously declined in probucol group. In addition, we found that the number of apoptotic cells in the CCH group and the probucol group was significantly higher than that in the sham group. Compared with the CCH group, the number of apoptotic cells in the probucol group was significantly reduced.

ROS is recognized as a key trigger for the activation of NLRP3 inflammasome, a vital protein complex that activates pyroptosis-related pathways. The inflammasome comprises NLRP3, ASC, and pro-Caspase-1. Upon activation, NLRP3 recruits ASC, which connects with pro-Caspase-1 to activate it into Caspase-1. Caspase-1 can then facilitate the secretion of inflammatory cytokines, interleukin-1β (IL-1β) and IL-18, by activating pro-IL-1β and pro-IL-18, thereby promoting a series of inflammatory responses [27]. In VD, pyroptosis leads to neuronal loss, damage, and brain function impairment. A clinical study [28] has manifested that inflammatory cytokines including IL-1β and IL-18 are elevated in the hippocampal tissues of VD patients. In addition, decreases in the expression levels of ASC, Caspase-1, and IL-1β/IL-18 have been shown to alleviate the cognitive impairment of gerbils after I/R [29], and ASC exacerbates ischemic neurological deficits and inflammatory reactions in an NLRP3-dependent manner. Moreover, the continuous release of inflammatory cytokines will continue to activate microglial cells, aggravate inflammatory responses, and further impair brain function. Microglial pyroptosis is also a pro-inflammatory programmed cell death, which contributes to the progression of Parkinson's disease and cognitive impairment in diabetic rats with CCH [5]. The hippocampus is an essential structure regulating learning and memory. There are a large number of microglial cells in the hippocampus, which can be activated by long-term ischemia and hypoxia. In this study, immunofluorescence staining and HE staining showed that probucol could attenuate microglial migration as well as neuronal damage. Similar findings were also reported by Tao Guo et al. [30] in a rat model of VD. In addition, WB assay results illustrated significant decreases in the protein expressions of NLRP3, ASC, and Caspase-1 and the secretion of pro-inflammatory cytokines in the hippocampus of rats in probucol group. Similar results were also obtained in microglial cells under lox-oxygen and low-glucose conditions.

Although the NLRP3 inflammasome is continuously studied in various diseases, the exact molecular mechanism leading to the activation of the NLRP3 inflammasome has yet to be fully elucidated. The three molecular mechanisms of NLRP3 inflammasome activation include increased ROS generation, lysosome destruction, and elevated K^+^ efflux [13]. Studies have found [31, 32] that Syk is an upstream kinase that activates the NLRP3 inflammasome. In addition, studies [15] have shown that NADPH oxidase-induced ROS is critical for NLRP3 inflammasome activation in macrophages, and ROS production has also been found to be mediated through Syk. In this study, we found that CCH can cause an increase in the expression of p-Syk, and Probucol can effectively inhibit the overexpression of p-Syk, ROS, NLRP3, ASC, and Caspase-1. Syk shows a high expression in microglial cells. In ischemic stroke and neurodegenerative diseases [33, 34], Syk can reduce the activation of microglial cells by down-regulating the expression of p-Syk and improve related symptoms by lowering the expression of p-Syk. Similar results were obtained in this study, that is, evident increases in the activation of microglial cells in the hippocampus of rats in CCH group and the protein expression of p-Syk were observed. Probucol could significantly decrease the expression of p-Syk, reduce the activation of microglial cells, and lower the expressions of NLRP3, ASC, and Caspase-1 in microglial cells, playing a neuroprotective role.

To sum up, this study demonstrates that probucol treatment can inhibit p-Syk expression, so as to reduce microglial activation, pyroptosis, and ROS production in the hippocampus of VD mice. While further research is still needed to investigate the role of Syk in VD, the presented results provide new insights into the mechanism of probucol in improving learning and memory in VD.

## Supplementary Materials

Supplementary Table 1
